# Machine learning approaches for predicting progression in hormone-sensitive prostate cancer patients

**DOI:** 10.3389/fonc.2026.1704671

**Published:** 2026-02-12

**Authors:** Bingyu Zhu, Haiyang Jiang, Chongjian Zhang, Qilin Wang, Libo Yang, Hong Yang, Ruiqian Li, Jun Li, Xusong Pang, Yufeng Zheng, Lingtao Yan, Yu Wang, Yu Bai

**Affiliations:** 1 Department of Urology, The Affiliated Chengdu 363 Hospital of Southwest Medical University, Chengdu, Sichuan, China; 2 Department of Urology I, The Third Affiliated Hospital of Kunming Medical University(Peking University Cancer Hospital Yunnan, Yunnan Cancer Hospital, Cancer Center of Yunnan Province), Kunming, Yunnan, China; 3 Department of Urology II, The Third Affiliated Hospital of Kunming Medical University (Peking University Cancer Hospital Yunnan, Yunnan Cancer Hospital, Cancer Center of Yunnan Province), Kunming, Yunnan, China

**Keywords:** ensemble learning, hormone-sensitive prostate cancer, machine learning, predictive model, prostate cancer progression

## Abstract

**Objective:**

Almost all hormone-sensitive prostate cancer (HSPC) cases eventually progress to castration-resistant prostate cancer (CRPC) following androgen deprivation therapy (ADT). This study aims to develop a machine learning (ML) model to predict the progression of HSPC patients. Additionally, we conducted statistical analyses on the dataset to identify significant features and clinical markers predictive of HSPC transitioning to CRPC.

**Methods:**

Data from 410 HSPC patients treated at Yunnan Cancer Hospital between 01/01/2017, and 31/05/2022, were analyzed. Predictive analyses were performed on a series of features observed during the patients’ initial visits. The primary ML methods employed were decision tree (DT), random forest (RF), XGBoost, artificial neural network (ANN), and support vector machine (SVM). Feature selection was conducted using a genetic algorithm (GA). The ML models were trained with an 80% training set and validated with a 20% test set. Model performance was evaluated using the area under the ROC curve (AUC), calibration plots, and learning curves to assess fit and calibration. Evaluation metrics included accuracy (ACC), precision (PRE), specificity (SPE), sensitivity (SEN), and F1 score.

**Results:**

Visualization of evaluation metrics was presented through confusion matrices and ROC curves. Ensemble learning methods, particularly RF and XGBoost, demonstrated the best model performance. RF achieved a score of 0.838 (95% CI:0.8324-0.902)on the training dataset and 0.817 (95% CI: 0.659 - 0.829) on the test dataset (AUC: 0.873, 95% CI:0.730-0.878). XGBoost achieved a score of 0.814 (95% CI:0.790-0.878) on the training dataset and 0.805 (95% CI:0.707-0.829) on the test dataset (AUC: 0.866, 95% CI:0.780-0.871). Calibration curves indicated good model calibration, and learning curves suggested no significant overfitting in both the training and test sets.

**Conclusion:**

Our findings demonstrate that ensemble learning methods, particularly RF, exhibit superior performance in predicting HSPC progression. This study represents a preliminary step toward a predictive tool, highlighting the potential of baseline clinical data for risk stratification. Future prospective studies with larger, multi-center cohorts are warranted to validate and refine this approach for possible clinical integration.

## Introduction

Prostate cancer ranks as the second most common malignancy in men worldwide, after lung cancer (1). Prognostic indicators such as Gleason score, prostate−specific antigen (PSA), and other serum markers have been shown to play important roles in assessing outcomes for prostate cancer patients (2). For instance, Ali Amin et al. (3) reported that a Gleason score of 4 + 3 = 7 is associated with an elevated risk of biochemical progression after radical prostatectomy. While clinical decision−making often relies on these established markers and scoring systems, the complexity of real−world practice means that some patients still experience disease progression or relapse during androgen−deprivation therapy (ADT). Such progression frequently leads to the development of castration−resistant prostate cancer (CRPC), which is linked to reduced quality of life and shorter survival (4–6). Therefore, preventing disease progression in prostate cancer patients is critical, underscoring the need for new technologies and methods to reliably predict progression.

Machine learning (ML), a branch of artificial intelligence (7), is increasingly employed in clinical settings for predictive analytics and optimizing management decisions. It has demonstrated promising results in areas such as diabetic retinopathy detection and breast−lesion diagnosis, highlighting its potential for broader clinical application (8). However, research on ML for prostate cancer prognosis remains limited, with most existing models focusing on survival outcomes. Among the few studies that address disease progression, one that monitored prostate cancer patients (9) developed logistic regression (LR), artificial neural network (ANN), and other models, evaluating them using the F1−score. Although all models yielded F1−scores below 0.6 due to certain limitations, the study still confirmed the feasibility of using ML to predict progression in this population.

The present study aims to leverage ML to build predictive models for hormone−sensitive prostate cancer (HSPC) patients receiving various non−surgical treatments. Specifically, we seek to forecast biochemical or radiographic progression within 18 months based on a range of biochemical indicators and clinical characteristics. Furthermore, we intend to identify independent risk factors for progression in HSPC patients using ML−based analysis. To this end, we collected data from HSPC patients diagnosed at the Department of Urology, Yunnan Cancer Hospital, and constructed five ML models: decision tree (DT), random forest (RF), eXtreme Gradient Boosting (XGBoost), ANN, and support vector machine (SVM).

## Methods

### Study population

This retrospective study comprised 410 patients diagnosed with hormone-sensitive prostate cancer (HSPC) who sought care at Yunnan Cancer Hospital between 01/01/2017, and 31/05/2022, with a follow-up deadline of 11/30/2023. The cohort included both non-metastatic and metastatic patients.The inclusion criteria were:

1. Histologically confirmed diagnosis of HSPC.
2. Presence of measurable lesions on imaging examinations (ultrasound, CT, MRI, bone scan, etc.).
3. Provision of complete medical records and follow-up data.
4. Receipt of treatment with luteinizing hormone-releasing hormone agonists (LHRHa) during the treatment course.

Exclusion criteria were:

1. Lack of histologically confirmed diagnosis of prostate cancer.
2. Presence of concurrent other malignancies.
3. Presence of systemic diseases affecting hematological indicators, such as blood disorders or immune system disorders.
4. Refusal or inability to complete clinical data collection or follow-up due to other reasons.
5. Presence of other severe organ diseases.

### Data collection

#### Patient characteristics

Patient characteristics encompassed age at diagnosis, TNM staging, Gleason score, testosterone level at initial diagnosis, prostate volume at initial diagnosis, fPSA, TPSA, and their ratio at initial diagnosis, high volume disease (HVD)(In the dataset, the feature is referred to as “tumor burden.”), presence of bone metastasis and visceral metastasis, alkaline phosphatase at initial diagnosis, treatment phase of hormone-sensitive disease, clinical data, and imaging data including ultrasound, CT, PET-CT, MRI, and whole-body bone scan.

HVD was defined as having visceral metastasis or bone metastasis ≥4 sites, with at least one site outside the spine or pelvis (10).

The primary endpoint of this study was defined as the time to castration-resistant prostate cancer (TTCRPC). Progression was determined based on sustained castrate serum testosterone levels (<1.7 nmol/L or <50 ng/dL) and meeting at least one of the following criteria: (1) PSA progression, defined as three consecutive rises in serum PSA (measured ≥1 week apart), with the second and third measurements each showing a >50% increase above the nadir (or an absolute increase >2 ng/mL if the nadir was <2 ng/mL); or (2) radiographic progression, defined as the discovery of new lesions on imaging, including either two or more new bone metastases on bone scan or any new soft−tissue lesions.For patients who did not exhibit PSA progression or radiographic progression, the last follow-up time served as the endpoint.

### Model development

#### Features and preprocessing

Our dataset encompasses 25 variables (referred to as features in machine learning), comprising 18 categorical variables and 7 continuous variables. Variables containing missing, erroneous, or ambiguous data were removed prior to analysis.

#### Feature selection

Given the large feature set and relatively limited sample size, feature selection was performed using the training set only, rather than the entire dataset. We employed Recursive Feature Elimination with Support Vector Machine (RFE−SVM), Least Absolute Shrinkage and Selection Operator (LASSO), genetic algorithm (GA), and grey wolf optimizer (GWO) (11–14) for this purpose. Our analysis revealed that models constructed using features selected by GA demonstrated superior performance.

**Figure 1:**
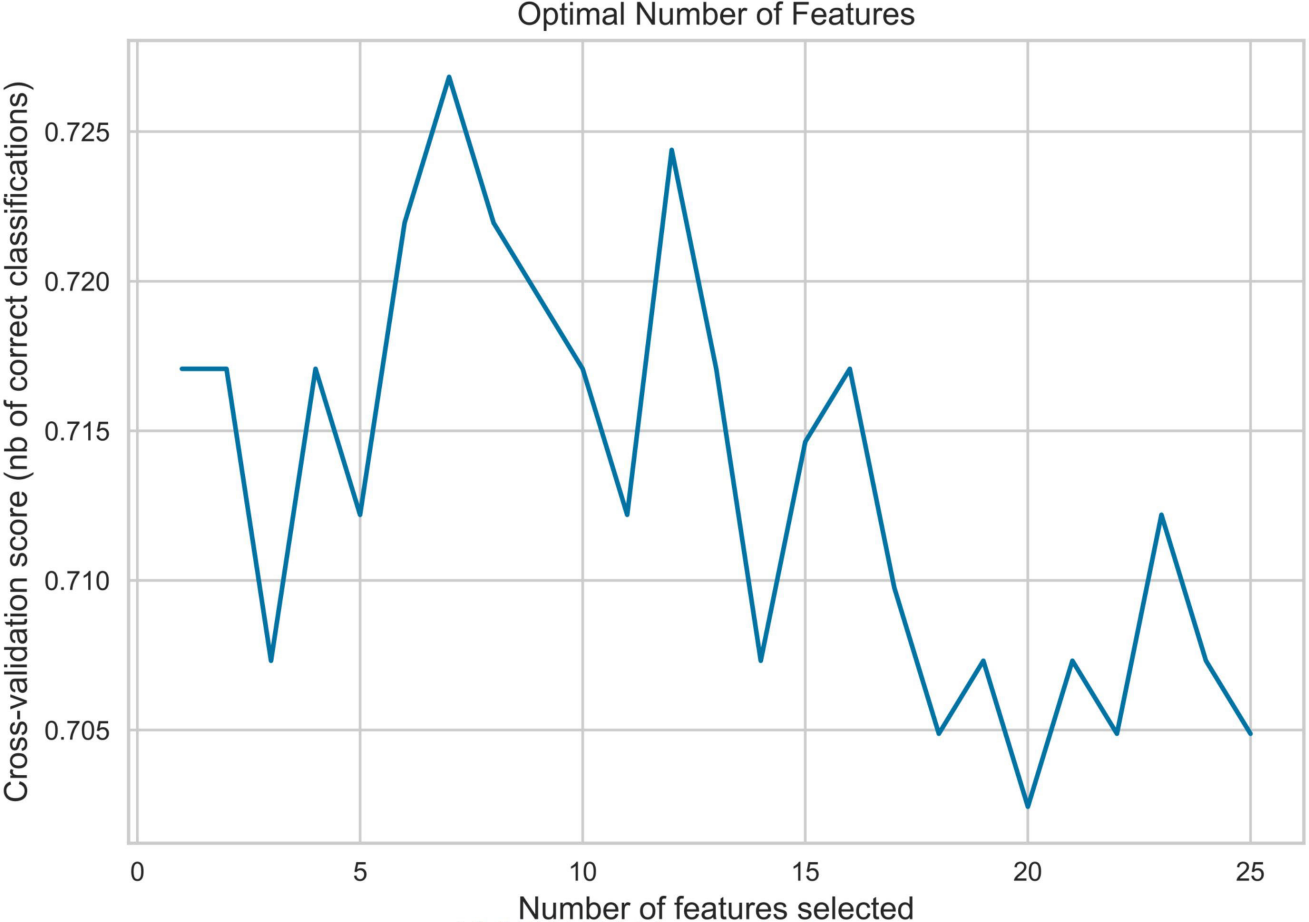
RFE-SVM feature selection.

**Figure 2:**
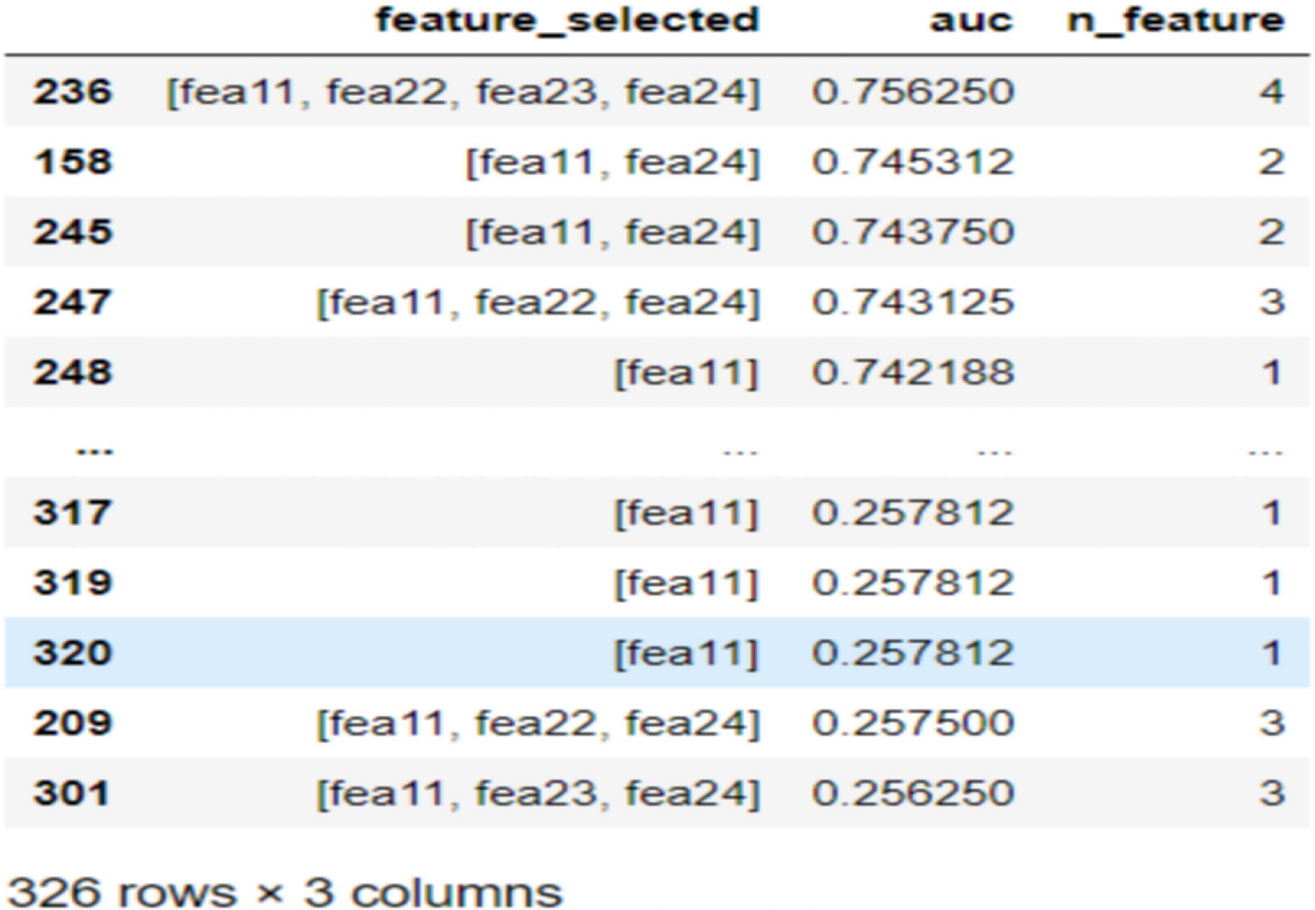
Lassonet feature selection.

Notably, during feature selection, some models incorporated the Synthetic Minority Over−sampling Technique (SMOTE) (15). To evaluate the effect of oversampling, we compared models trained on features derived with and without SMOTE. The improved performance observed with SMOTE likely stems from its ability to mitigate class imbalance, thereby helping the model learn the minority class more effectively. However, during final model construction, we did not apply SMOTE, in order to assess performance on the original, imbalanced data and to avoid potential bias introduced by oversampling. Thus, while SMOTE was used in the feature−selection phase, the original sample distribution was retained for model building to ensure objective and reliable findings. Feature−selection plots for RFE−SVM and LassoNet are shown in **Figures 1**, **2**, respectively.

### Machine learning model strategy

#### Model description and rationale

The final outcome samples were partitioned into an 80% training set (n=328) and a 20% test set (n=82). We employed five distinct machine learning models, namely DT, RF, XGBoost, ANN, and SVM, to discern patient outcomes. The selection of these models was based on the following rationale: ensemble learning, epitomized by decision trees, exhibits robust classification power. RF amalgamates the outputs of multiple classifiers via majority voting or averaging to yield the final result, commonly applied to classification and regression tasks. It constructs multiple decision trees by randomly selecting features and samples, then merges them to make the final prediction. RF aggregates predictions from multiple decision trees through majority voting or averaging, commonly used for classification and regression tasks. By constructing trees from randomly selected features and samples, RF reduces overfitting and generally shows good generalization ability (16). XGBoost improves model performance by iteratively training decision trees and optimizing an objective function via gradient descent, with enhancements in feature handling, regularization, and computational efficiency, often leading to higher predictive accuracy (17). DT uses a tree-like structure to split the dataset based on feature thresholds, assigning outcomes through branching rules. It is intuitive, interpretable, adaptable to diverse data types, and requires minimal data preprocessing (18). ANN mimics biological neural networks with layered neurons, learning mappings between inputs and outputs to recognize patterns and make predictions. While capable of modeling complex relationships, ANN typically requires substantial data and computational resources for training (19). SVM identifies an optimal hyperplane that maximally separates classes while maximizing the margin to the nearest data points. It performs well in high-dimensional spaces and on complex datasets, exhibiting strong generalization (20).

#### Data preprocessing and model training strategy

Data Standardization: Prior to model training, the dataset was partitioned. For machine learning models sensitive to the scale of input features, specifically the SVM and ANN, all continuous variables (e.g., TPSA, fPSA, LDH, ALP) were standardized using Z-score normalizationvia StandardScaler from the scikit-learn library. Tree-based models (RF, XGBoost, DT) are invariant to feature scaling;therefore, standardization was not applied to the data subsets used for these models.

Parameter tuning primarily relied on ten-fold cross-validation and grid search to determine hyperparameters. To mitigate overfitting, we implemented the Early Stopping strategy to refine the obtained functions. To ensure model stability, we consistently set the random seed to 42. Parameter adjustments are detailed in the **Supplementary Materials**. Subsequent model evaluations will be conducted using these parameters as a reference. Traditional statistics serve as a supplement in this study. Furthermore, we employed the bootstrap method to conduct 300 resamplings for internal validation of model scores and AUC.

### Statistical analysis

#### Data distribution and group comparisons

We modeled the data features of these patients and conducted the aforementioned parameter adjustments. Different subsets of patient features and baseline data are detailed in **Table 1** and **Supplementary Materials**. We utilized the Shapiro-Wilk test in Python software to evaluate the normality of continuous variables. For normally distributed data, we applied the independent samples t-test and reported statistics as “mean ± standard deviation.” For non-normally distributed data, we employed the Wilcoxon rank-sum test and reported statistics as “median (25%–75%) [M (P25-P75)].” Count and ordinal data were presented as frequency (%), while differences between groups for binary variables were assessed using the chi-square test.

**Table 1:** Statistical description of patients’ baseline data.

Baseline data	Progress group(n=140)	Un-progress group(n=270)	Statistic	P-value
age	68±8	69±7.2	-0.629	0.529
Symptoms of obstruction appear			14.783	<0.001
appear	90 (64.29)	220(81.48)		
Not yet appeared	50 (35.71)	50 (18.52)		
Symptoms of hematuria appear			0.135	0.713
appear	14 (10.00)	24 (8.89)		
Not yet appeared	126(90.00)	246(91.11)		
Whether nodules are touched			4.229	0.0397
touched	88 (62.86)	141(52.22)		
Not touched	52 (37.14)	129(47.77)		
Pathological type			1.249	0.264
Adenocarcinoma	139(99.29)	264(97.78)		
Non-adenocarcinoma	1 (0.71)	6 (2.22)		
Gleason score			19.102	<0.0001
<8	21 (15)	96 (35.56)		
≥8	119(85)	174(64.44)		
Prostatic volume(cm)	97.8 (69.228~144.576)	86.913 (61.413~129.419)	1.480	0.139
fPSA(ng/ml)	28.08 (13.7~50)	19.4 (7.83~50)	7.892	<0.0001
Initial TPSA(ng/ml)	592.1 (191.325~1839.5)	126.15 (24.7675~482.75)	7.949	<0.0001
First diagnosis of bone metastasis			52.256	<0.0001
appear	129(92.14)	155(57.41)		
Not yet appeared	11 (7.86)	115(42.59)		
Initial diagnosis of visceral metastasis			0.009	0.923
appear	16 (11.43)	30 (11.11)		
Not yet appeared	124(88.57)	240(88.89)		
Tumor burden			60.809	<0.0001
High load	126(90.00)	138(51.11)		
Low load	14 (10.00)	132(48.89)		
T-stage			24.649	<0.0001
T1-T2	11 (7.86)	79 (29.26)		
T3-T4	129(92.14)	191(70.74)		
N-stage			31.296	<0.0001
N0	30 (21.43)	135(50.00)		
N1	110(78.57)	135(50.00)		
M-stage			53.419	<0.0001
M0	8 (5.71)	108(40.00)		
M1	132(94.29)	162(60.00)		
LDH(U/L)	211.5 (185.5~268)	188 (164.5~217.5)	5.454	<0.0001
Alkaline phosphatase( U/L)	178.5 (100~397)	97 (76~170.75)	6.159	<0.0001
Testosterone levels at first diagnosis(ng/dL)	428.86 (281.235~548.225)	437.040 (333.9~526.075)	-0.264	0.792

Significance level: α=0.05, with P<0.05 indicating statistical significance.

The selection of an appropriate evaluation metric is crucial for assessing the effectiveness of classification models. While various metrics are available, the F1 score is preferred due to its ability to balance precision and recall metrics, offering a comprehensive assessment of a model’s performance. The F1 score strikes a delicate balance between maximizing the identification of positive instances (high recall) and ensuring the accuracy of the identified positives (high precision). In our investigation, we evaluated several state-of-the-art (SOTA) models, including DT, XGBoost, RF, ANN, and SVM. These models were assessed across multiple performance metrics such as accuracy rate (ACC), sensitivity (SEN), specificity (SPE), precision (PRE), F1-score, and the area under the ROC curve (AUC).

The Python version utilized for the entire machine learning process was 3.10.9.

The Shapiro-Wilk test is primarily employed to determine whether a sample originates from a normally distributed population. Its null hypothesis posits that the data is derived from a normal distribution. If the p-value is less than the significance level (typically set to 0.05), the null hypothesis is rejected, indicating that the data does not stem from a normal distribution (21). The Wilcoxon rank-sum test compares differences between two related or paired samples, with its null hypothesis being that the median difference between two related samples is zero. If the p-value is less than the significance level, the null hypothesis is rejected, indicating a significant difference between the two samples (22). The chi-square test evaluates disparities between observed and expected frequencies, commonly employed for testing associations between categorical variables. The null hypothesis asserts that there is no difference between observed and expected frequencies. If the p-value is less than the significance level, the null hypothesis is rejected, indicating a significant difference between observed and expected frequencies (23).

#### Heatmap analysis

Prior to model establishment, we employed a heatmap to visualize the correlations between different features. A heatmap serves as a data visualization tool to illustrate relationships within a matrix dataset. The color intensity or numerical value of the heatmap reflects the degree of relationship between features. High collinearity in the heatmap indicates strong correlations between feature changes, suggesting that alterations in one feature are often accompanied by corresponding changes in another feature (24).

#### Model performance

SHAP is a global explanation method grounded in the Shapley value principle from cooperative game theory, utilized to explain the contribution of each feature to each prediction in a model. It assesses the influence of each feature by considering all possible feature combinations, thereby furnishing a global explanation. SHAP explanations serve to elucidate the rationales behind individual predictions or to comprehend the behavior of the entire model. LIME, on the other hand, is a local explanation method designed to elucidate the rationales behind individual predictions. It accomplishes this by generating a local neighborhood in the input space and fitting a simple explanation model. While the explanation model generated by LIME is effective within the local neighborhood, its applicability may not extend globally. The advantage of LIME lies in its simplicity and ease of understanding, rendering it particularly suitable for explaining black-box models, such as deep learning models (25). FI, as employed in this study, primarily pertains to tree models and evaluates the influence of each feature on prediction outcomes. Understanding feature importance facilitates comprehension of the model’s reliance on different features, thereby assisting in feature selection, model optimization, and result interpretation (26).

## Results

During the study period (01/01/2017 to 31/05/2022), 410 HSPC patients meeting inclusion/exclusion criteria were enrolled, with 140 experiencing disease progression and 270 progression-free. No significant differences existed in age (progressed: mean 68 ± 8 years vs. non-progressed: 69 ± 7.2 years; p=0.529) or testosterone levels (progressed: median 428.86 ng/dL, IQR 281.24–548.23 vs. non-progressed: 437.04 ng/dL, IQR 333.9–526.08; p=0.792). Progressed patients exhibited significantly higher-risk features: lower urinary obstruction prevalence (64.29% vs. 81.48%; p<0.001), higher palpable nodule rates (62.86% vs. 52.22%; p=0.0397), greater Gleason score ≥8 frequency (85% vs. 64.44%; p<0.0001), elevated fPSA (median 28.08 ng/mL, range 13.7–50 vs. 19.4 ng/mL, range 7.83–50; p<0.0001), and markedly higher initial TPSA (median 592.1 ng/mL, range 191.33–1839.5 vs. 126.15 ng/mL, range 24.77–482.75; p<0.0001). Metastatic burden was significantly greater in progressed patients: bone metastasis (92.14% vs. 57.41%; p<0.0001), high-volume disease (90% vs. 51.11%; p<0.0001), advanced T-stage (T3-T4: 92.14% vs. 70.74%; p<0.0001), nodal involvement (N1: 78.57% vs. 50%; p<0.0001), and distant metastasis (M1: 94.29% vs. 60%; p<0.0001). Biomarker analysis revealed significantly increased LDH (median 211.5 U/L, IQR 185.5–268 vs. 188 U/L, IQR 164.5–217.5; p<0.0001) and ALP (median 178.5 U/L, range 100–397 vs. 97 U/L, IQR 76–170.75; p<0.0001) in the progressed group. No significant differences were observed in hematuria (10.00% vs. 8.89%; p=0.713), non-adenocarcinoma subtypes (0.71% vs. 2.22%; p=0.264), or visceral metastasis distribution (11.43% vs. 11.11%; p=0.923). This analysis identifies tumor aggressiveness markers (Gleason score, TPSA), high-volume disease, and advanced staging as core progression drivers, whereas age, testosterone, and certain local symptoms show limited predictive value.

**Figure 3:**
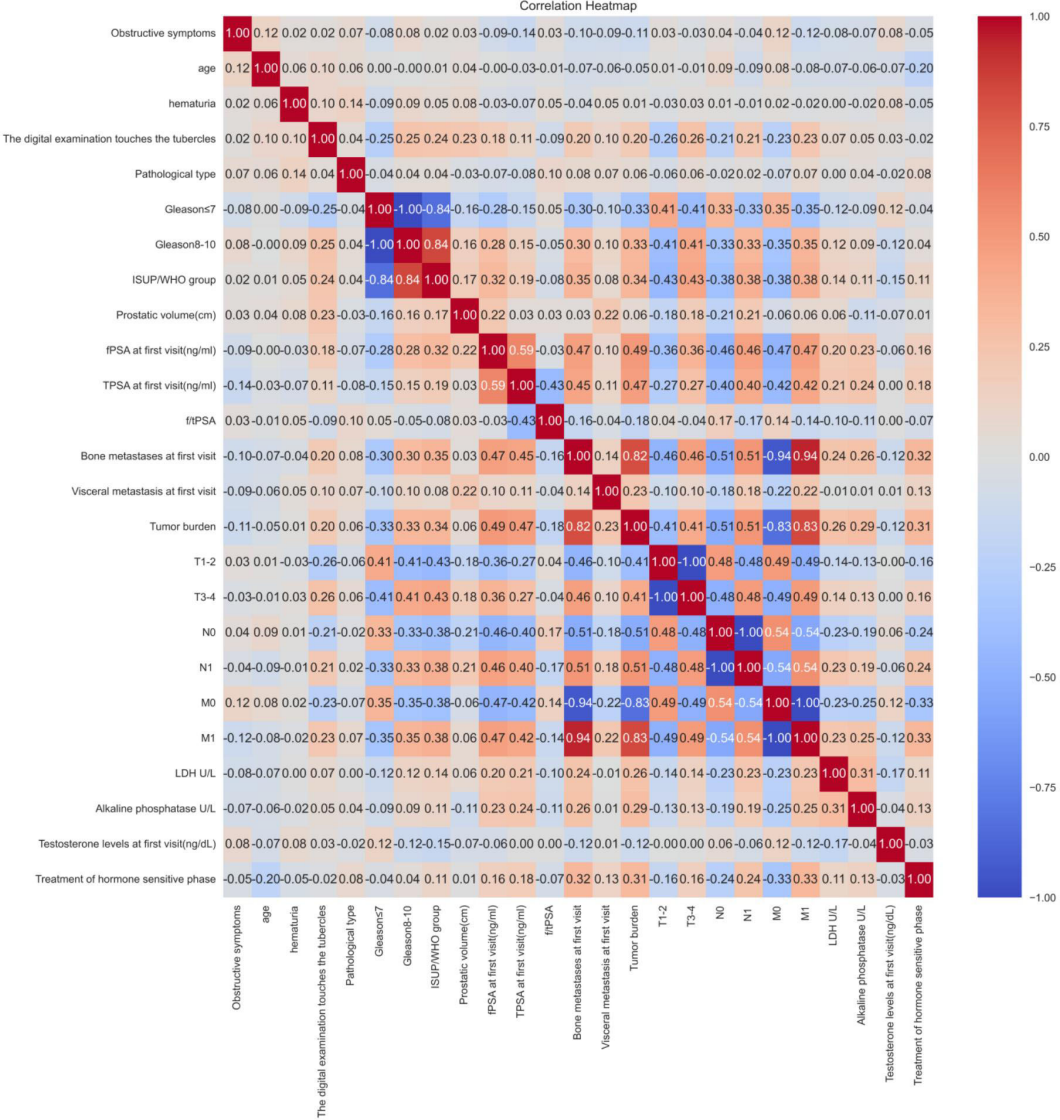
Heatmap of database.

The heatmap visualization is presented in **Figure 3** It is noteworthy that several pairs of features exhibit strong negative or positive correlation coefficients, as detailed in the Results section.

### Model performance

We applied the trained models to the test dataset. Among the models, ensemble learning methods, represented by RF and XGBoost, exhibited the best performance. RF achieved a score of 0.838 (95% CI: 0.834 - 0.902) on the training dataset and 0.817 (95% CI: 0.659 - 0.829) on the test dataset (AUC: 0.873, 95% CI: 0.730 - 0.878). XGBoost achieved a score of 0.814 (95% CI: 0.790 - 0.878) on the training dataset and 0.805 (95% CI: 0.707 - 0.829) on the test dataset (AUC: 0.866, 95% CI: 0.780 - 0.871).

The performance of other machine learning models on the training and test datasets are as follows: ANN achieved a score of 0.774 (95% CI: 0.726 - 0.840) on the training dataset and 0.720 (95% CI: 0.603 - 0.775) on the test dataset (AUC: 0.745, 95% CI: 0.716 - 0.813). DT achieved a score of 0.823 (95% CI:0.805 - 0.881) on the training dataset and 0.780 (95% CI: 0.585 - 0.817) on the test dataset (AUC: 0.844, 95% CI: 0.616 - 0.865). SVM achieved an accuracy of 0.750 (95% CI: 0.729 - 0.822) on the training dataset and 0.671 (95% CI: 0.622 - 0.720) on the test dataset (AUC: 0.749, 95% CI: 0.689 - 0.767).

**Figure 4:**
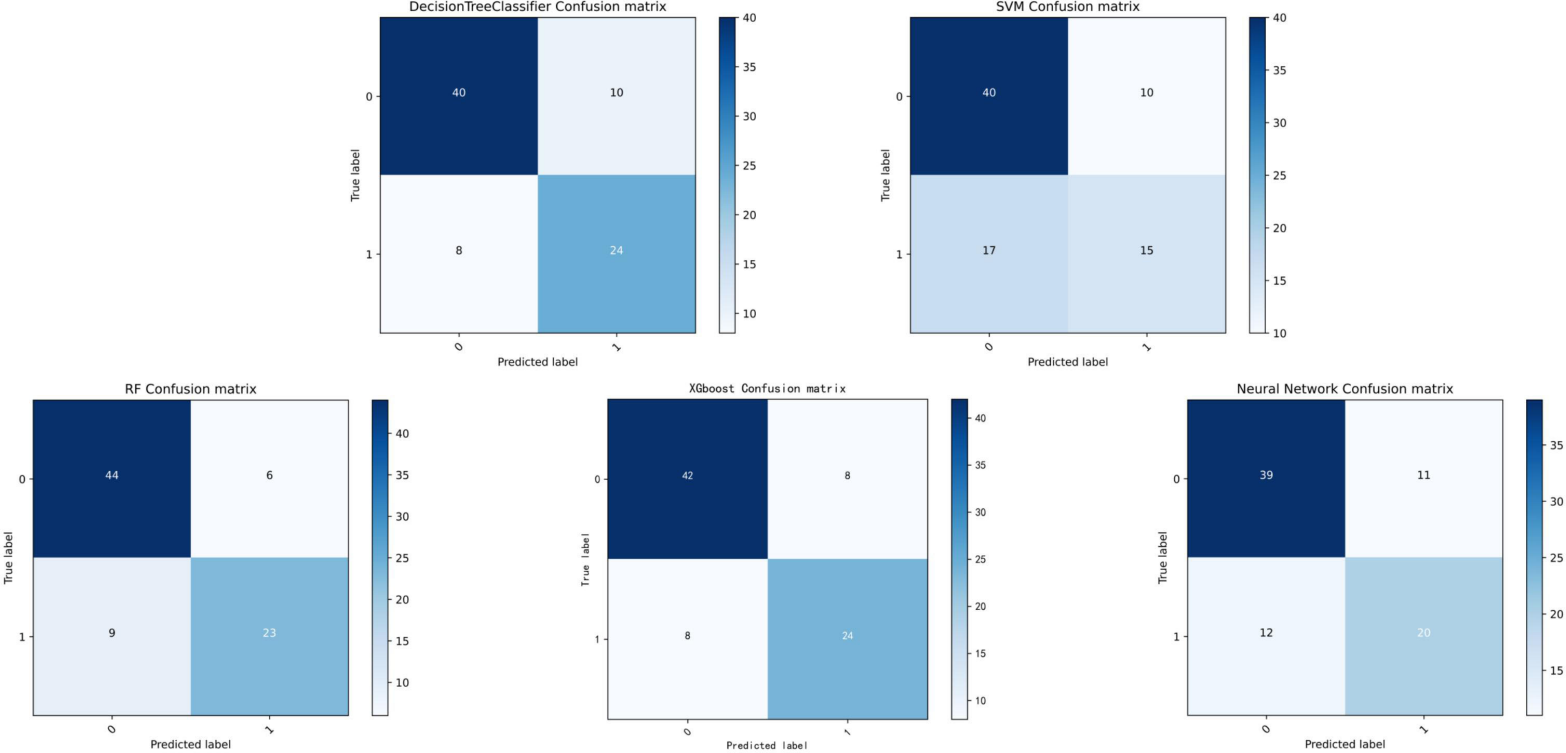
Confusion matrix of each model.

**Figure 5:**
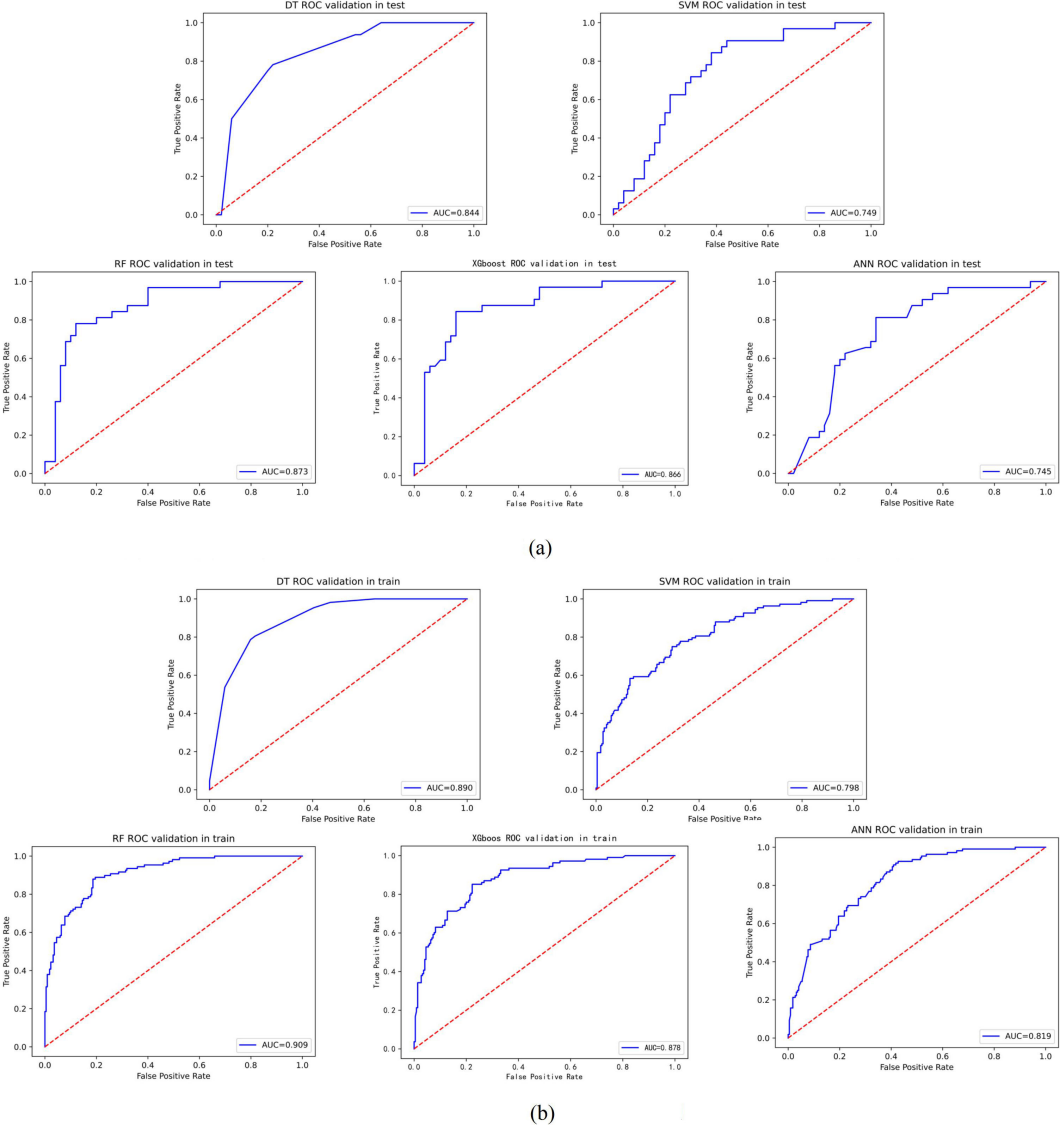
ROC curve of each model. **(A)** ROC curves in test of each model. **(B)** ROC curves in train of each model.

The performance metrics for all models are summarized in **Table 2**, with confusion matrices and ROC curves shown in **Figures 4**, **5A, B**. **6A, B** presents the learning curves for each model (cv=10), where the red and blue lines represent the average scores of the models on the training and test datasets as the sample size changes, respectively. The red and blue areas represent the standard deviation of the models. The AUC values for the training and test datasets are comparable, and the learning curves show no significant overfitting. **Figure 7** shows the calibration curves for each model. Calibration curves and the Brier score are essential metrics for evaluating the accuracy and reliability of machine learning model predictions. In an ideally calibrated model, the predicted probabilities align closely with observed probabilities, as represented by a calibration curve near the 45-degree reference line. This calibration characteristic is particularly important in clinical applications, where predictive accuracy directly impacts the soundness of clinical decisions. The Brier score further quantifies the error between predicted probabilities and actual outcomes, with lower scores indicating predictions that closely match observed frequencies, thereby enhancing the model’s reliability in real-world scenarios. In this study, the Brier score was selected as a key evaluation metric to comprehensively reflect the model’s predictive capability.Generally, a Brier score of 0 indicates perfect prediction, a score below 0.1 is excellent, 0.1-0.25 is good, and above 0.25 indicates the model needs improvement. Our models have Brier scores between 0.1 and 0.25, except for the SVM model, which exceeds 0.2, indicating good calibration for our models.

**Table 2:** Confusion matrix metrics for each model.

	accuracy rate (ACC)	sensitivity(SEN) or regression rate(Recall)	specificity (SPE)	precision(PRE)	f1-score	The area under the ROC curve (AUC)
DT	0.780	0.750	0.8	0.706	0.727	0.844
XGboost	0.805	0.750	0.840	0.750	0.750	0.866
RF	0.817	0.719	0.880	0.793	0.754	0.873
ANN	0.720	0.625	0.780	0.645	0.635	0.745
SVM	0.671	0.469	0.8	0.6	0.526	0.749

**Figure 6:**
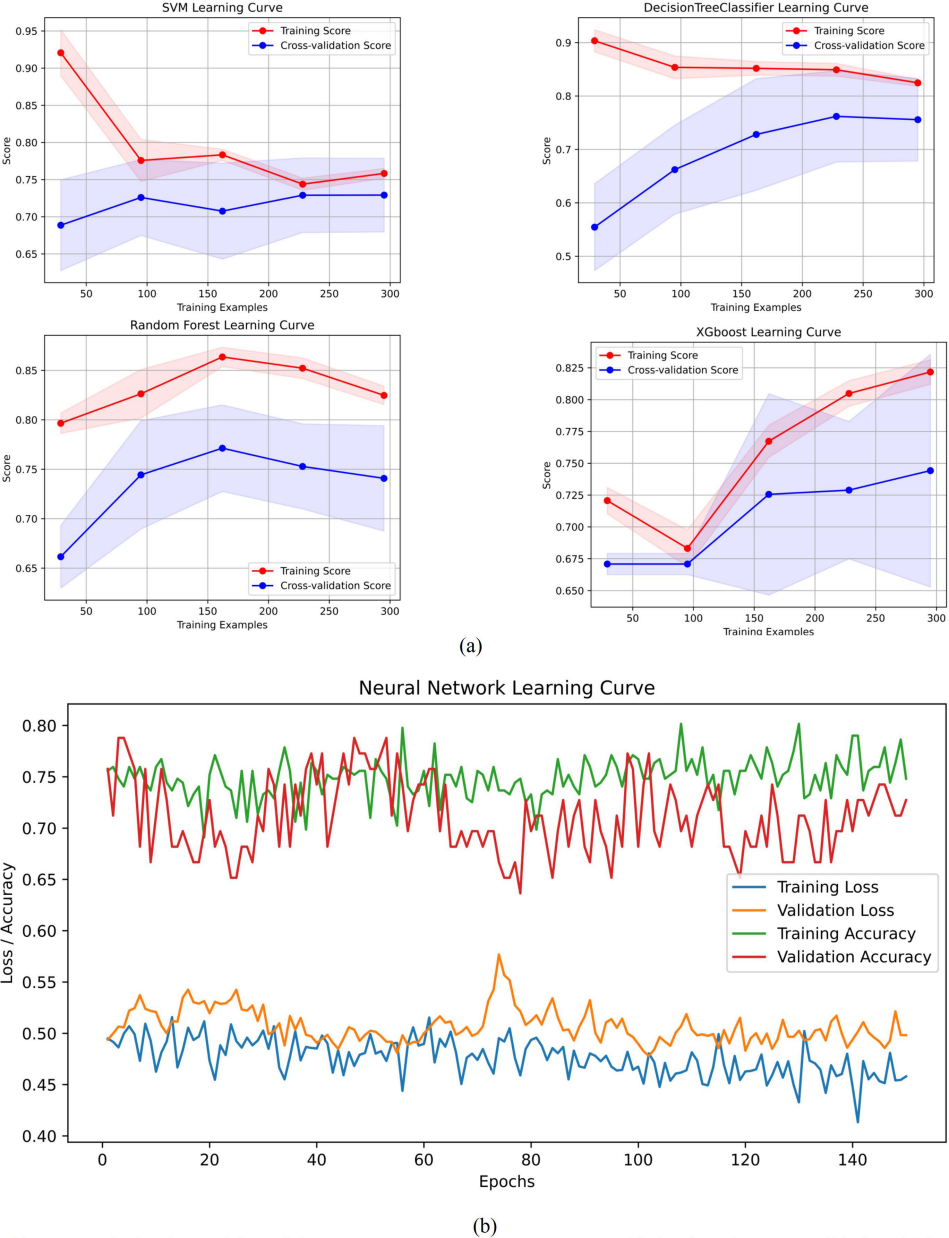
Learning curves for each model. **(A)** Learning curves excluding ANN. **(B)** Learning curve of ANN.

**Figure 7:**
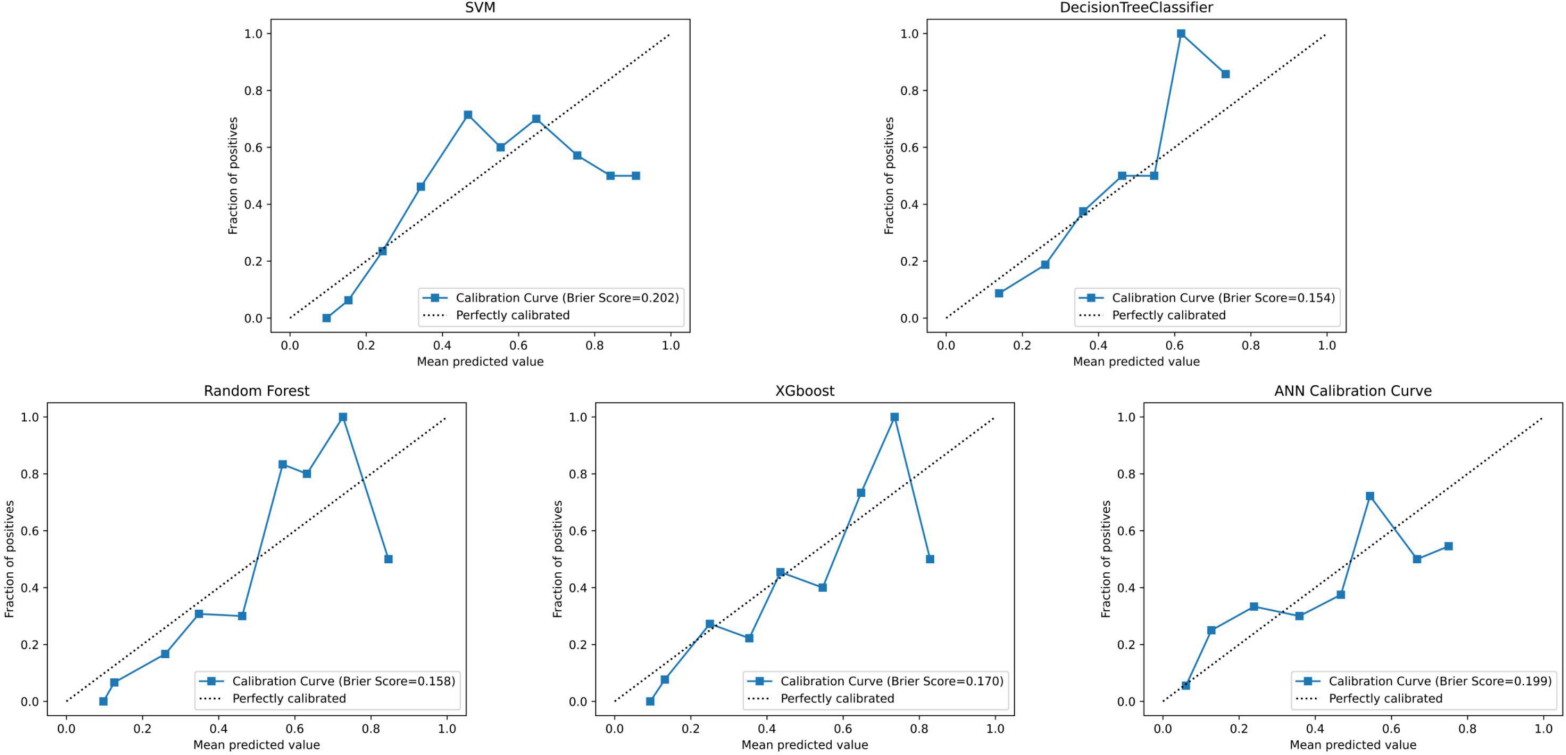
Calibration curves for each model.

Among the evaluated models, XGBoost demonstrated exceptional capability with an F1 score of 0.750. The F1 score, which combines precision and recall, provides a detailed assessment of the model’s classification ability. This superior performance underscores XGBoost’s effectiveness in balancing the maximization of true positive identification and the minimization of false positives.

### Importance analysis

**Figure 8:**
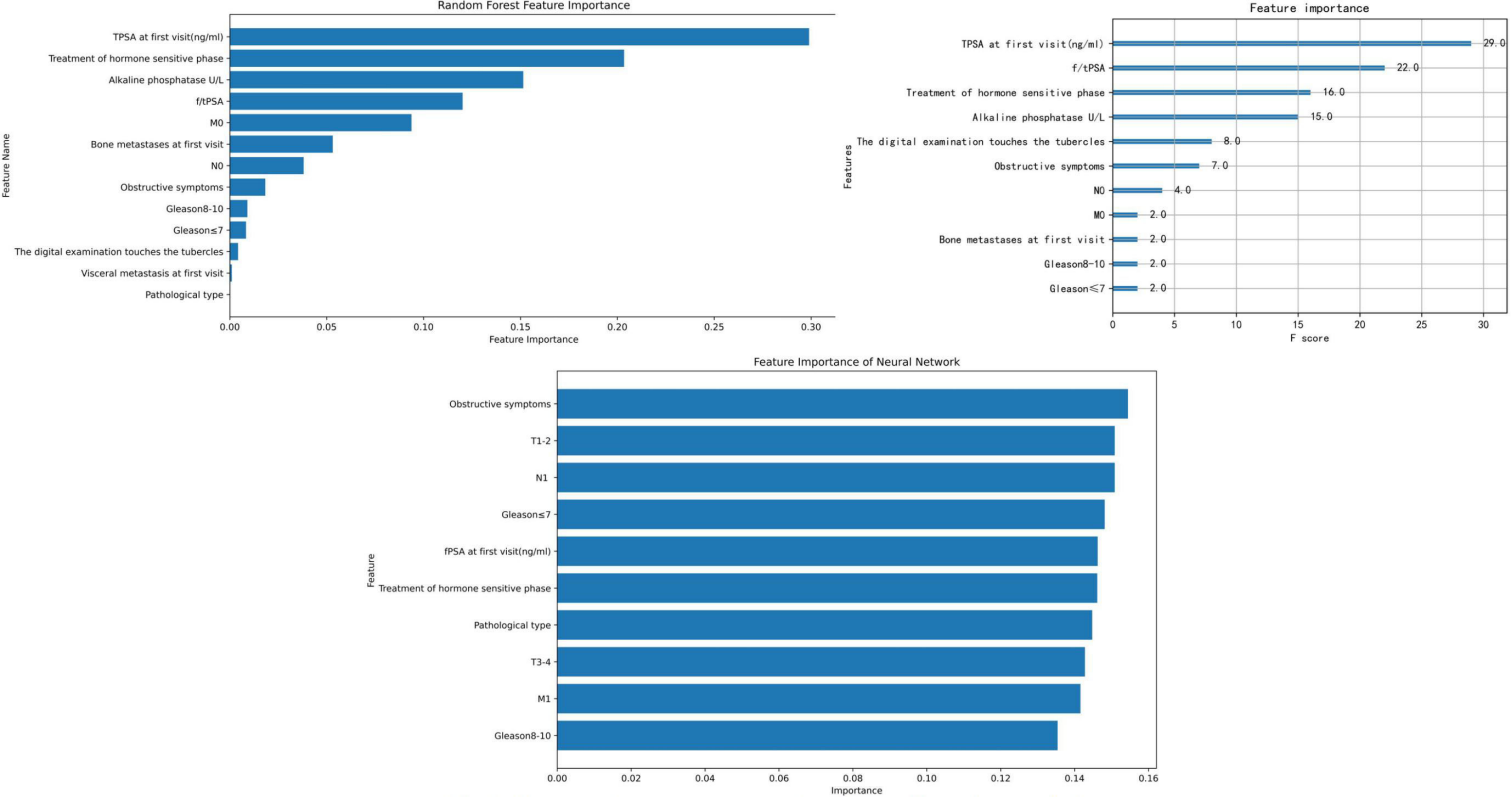
Feature importance selection of each model.

**Figure 9:**
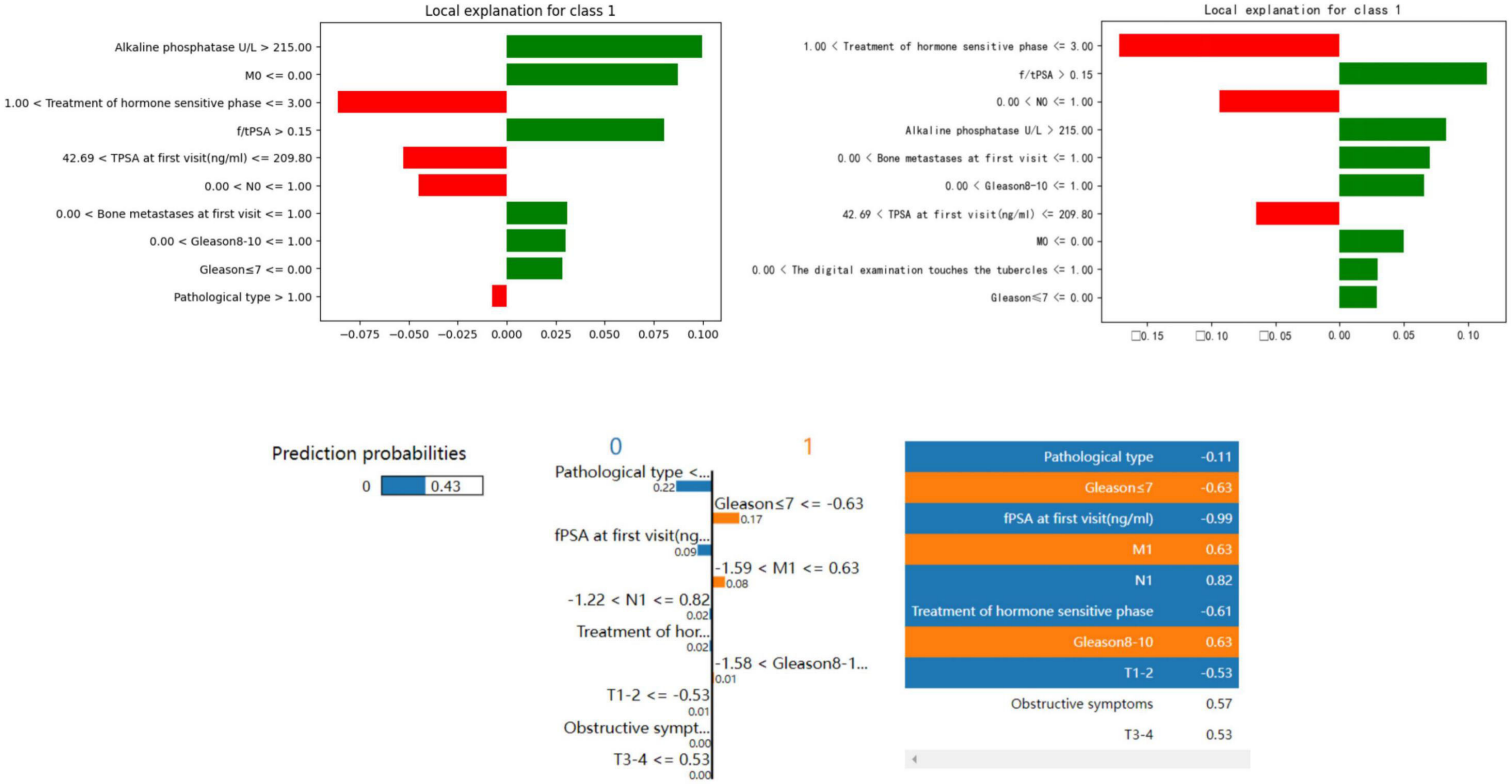
LIME for each model.

**Figure 10:**
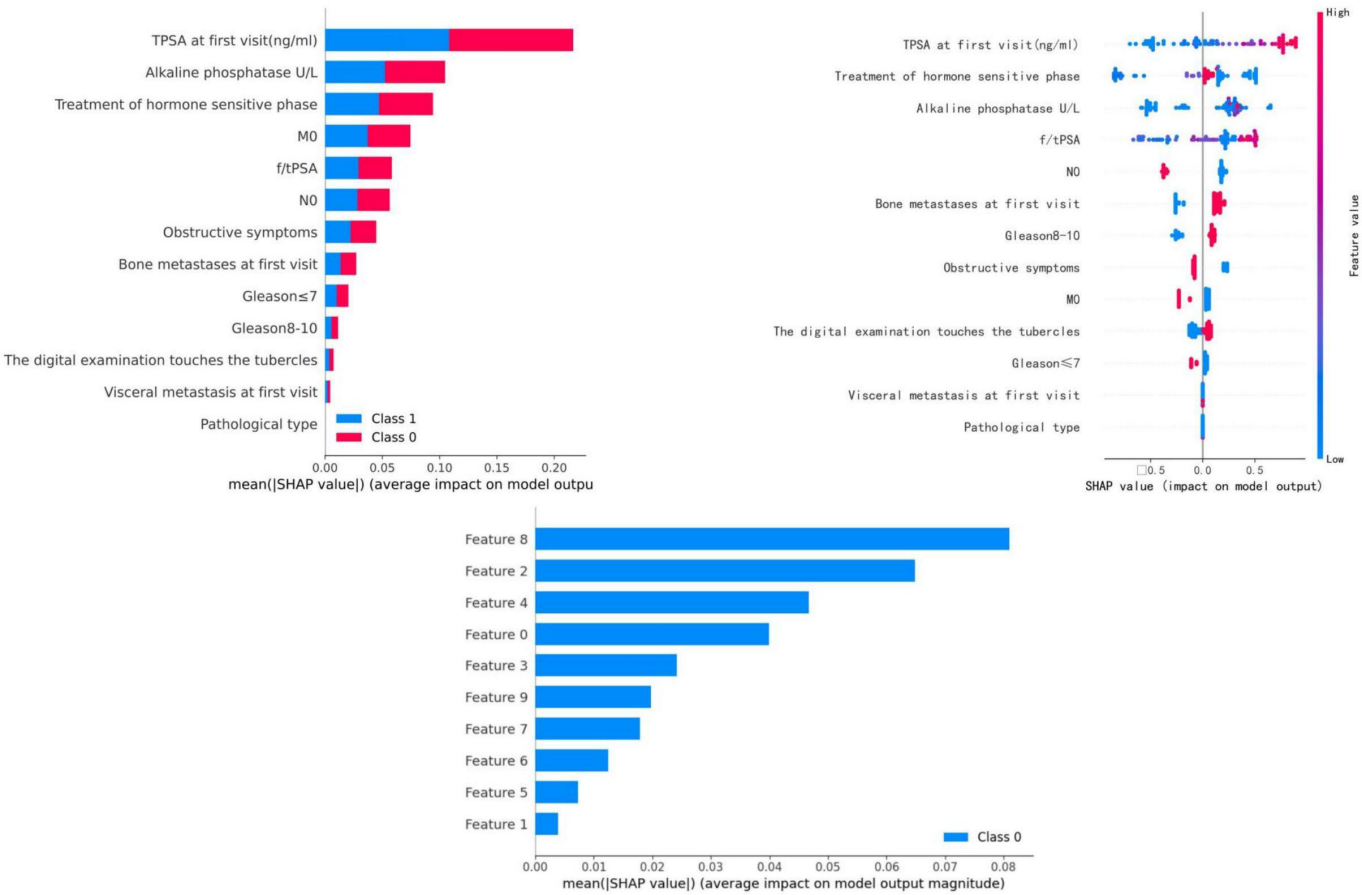
SHAP for each model.

We selected RF, XGBoost, and ANN for feature importance visualization. The feature importance (FI) for these three models is shown in **Figure 8**, LIME visualizations in **Figure 9**, and SHAP visualizations in **Figure 10** In the RF model’s FI, total prostate-specific antigen (TPSA), treatment of the hormone-sensitive phase, and alkaline phosphatase were the most important features. In XGBoost, TPSA, the ratio of free to total PSA (f/tPSA), treatment of the hormone-sensitive phase, and alkaline phosphatase were the most important features. In ANN, obstructive symptoms, T1-T2 staging, N1, and Gleason score ≤7 were the most important features.

In LIME visualizations, treatment of the hormone-sensitive phase, f/tPSA, and N0 were the most important features for XGBoost; alkaline phosphatase, M0, and treatment of the hormone-sensitive phase were the most important for RF; while pathological type, Gleason score ≤7, fPSA, and M1 were the most important for ANN.

In SHAP visualizations, TPSA, alkaline phosphatase, treatment of the hormone-sensitive phase, M0, and f/tPSA were the most important features for RF. For XGBoost, TPSA, treatment of the hormone-sensitive phase, and alkaline phosphatase were the most important features. For ANN, M1, Gleason score ≤7, fPSA, and obstructive symptoms were identified as the most important features.

## Discussion

This study systematically applied ML techniques—including DT, XGBoost, RF, ANN, and SVM—to analyze the initial diagnostic data of 410 HSPC patients treated at Yunnan Cancer Hospital (01/01/2017–31/05/2022). Our modeling investigated associations between clinical indicators, therapeutic approaches, and disease progression outcomes. Comparative performance evaluation identified RF as the most robust model across key metrics, demonstrating considerable potential for clinical risk stratification.

RF outperformed other models in accuracy (0.817 vs. 0.671–0.805), AUC (0.873 vs. 0.745–0.866), and specificity (0.880 vs. 0.780–0.840), indicating a superior ability to correctly identify non-progressing patients—a critical feature for confidently excluding low-risk cases. XGBoost approached RF in precision (0.750) and AUC (0.866) but exhibited lower sensitivity (0.750) and F1-score (0.750), suggesting suboptimal sample balance despite its utility in high-risk screening. Traditional models showed certain limitations: DT achieved a moderate AUC (0.844) but suffered from low precision (0.706) and F1-score (0.727), reflecting sensitivity to noise; SVM displayed markedly imbalanced sensitivity (0.469) and F1-score (0.526), likely due to linear kernel constraints. ANN underperformed in AUC (0.745) and specificity (0.780), potentially constrained by parameter optimization with a limited sample size, though its ability to capture complex feature interactions merits further investigation in larger studies.

All models underwent rigorous multidimensional validation. RF demonstrated stable convergence in learning curves without significant overfitting or underfitting and achieved a favorable Brier score (0.158 vs. ANN’s 0.199 and SVM’s 0.202) in calibration analysis. Bootstrap internal validation confirmed the generalizability of RF, with test-set accuracy of 0.817 (95% CI: 0.659–0.829) and AUC of 0.873 (95% CI: 0.730–0.878), showing minimal performance gap compared to the training set (accuracy 0.838, 95% CI: 0.834–0.902). These results demonstrate that ML models—particularly the ensemble-based RF—can reliably quantify progression risk in HSPC using baseline clinical and biochemical data. Future integration of dynamic follow-up data and multimodal imaging features may further enhance real-world generalizability and clinical decision support.

ML has attracted considerable attention in clinical settings for its ability to predict and analyze diseases (27). Applying ML methods to predict progression in prostate cancer is crucial for improving predictive accuracy and enabling personalized risk assessment. Traditional clinical decision-making often relies on empirical experience and limited statistical models, which may not effectively integrate complex biological data—such as gene expression profiles, imaging features, and pathology reports—into prognostic evaluations (9). By training predictive models, ML facilitates high-accuracy risk assessment and progression forecasting, empowering clinicians to develop personalized treatment strategies (8). Shinpei Saito et al. (28) used ML to predict outcomes in metastatic prostate cancer patients undergoing ADT, developing an RF model that achieved a C-index of 0.85 after incorporating time-series modeling. However, their study was limited by a dataset of only 310 cases and focused solely on PSA progression. Another study aiming to predict prostate cancer-specific mortality using the SEER database developed a Survival Quilts model, which differs from our approach (29). In contrast, our study incorporated a broader set of clinical data—including hormone levels, prostate volume, and obstructive symptoms alongside tumor markers and Gleason scores—making it more aligned with clinical practice. Furthermore, we utilized learning curves, calibration curves, and bootstrap internal validation to ensure model accuracy and mitigate overfitting, thereby enhancing the robustness of our models. It is noteworthy that while the application of machine learning in prostate cancer prognosis is increasing, related research has primarily concentrated on single-modality data such as genomics to explore the molecular mechanisms of CRPC (30, 31). In contrast, studies specifically dedicated to predicting the transition from HSPC to CRPC using multidimensional clinical data remain relatively scarce. Our study attempts to address this gap. Shifting focus from data modality to data source, our work also differs importantly from studies based on large public databases (e.g., SEER). For instance, Tang et al. (32) constructed a high-discrimination model (AUC >0.9) using SEER data, but its predictive endpoints are macro-level outcomes such as prognostic survival rates. Such endpoints are highly compatible with registry data dominated by categorical variables. However, predicting the progression from HSPC to CRPC is a more nuanced clinical problem, whose predictive power likely depends on continuous laboratory indicators that are often missing or simplified in SEER. Therefore, this study utilizes more in-depth data closer to real-world clinical scenarios to specifically address this issue, forming a strong complement to macro-level predictive models. This comparison also points the way for our future work: attempting to externally validate and calibrate baseline models built on large databases using the in-depth clinical data from our center may yield predictive tools that are both statistically powerful and aligned with clinical reality.

To clarify the relative importance of factors influencing patient progression, we applied feature importance (FI), SHAP, and LIME methods to interpret the models. After model construction, we observed that pathological type and Gleason score ranked relatively low in FI, rising in importance only in the LIME analyses for RF and ANN. This suggests that, in our models, prostate cancer progression may not be strongly associated with pathological type or Gleason score—a finding that contrasts with the prevailing consensus among prostate cancer experts (33). To explore this discrepancy, we used heatmaps and found low correlation coefficients (0.2–0.4) between Gleason score and TNM stage or PSA levels, and even lower for pathological type. This indicates limited collinearity between these features and others in our dataset. The reasons for the subdued influence of Gleason score and pathological type in our models remain unclear and warrant further investigation.

Despite these discrepancies, the interpretability methods yielded valuable insights. In FI, SHAP, and LIME analyses, treatment during the hormone-sensitive phase, PSA, and alkaline phosphatase consistently ranked as the most important features in the ensemble learning models. Shinpei Saito et al. (28) also identified PSA as the most significant feature in their model. Both PSA and treatment in the hormone-sensitive phase are well-established prognostic factors in prostate cancer, aligning with expert consensus (34–38).

Our study further corroborates the impact of bone metastases on prostate cancer progression, as reflected in both traditional statistical analysis (alkaline phosphatase, p < 0.0001) and model interpretations. These findings carry important clinical implications. Japanese researchers have similarly highlighted the association between prostate cancer metastasis and patient prognosis (39). However, there is currently no treatment specifically targeting bone metastases in prostate cancer. In limited available research, Stella D’Oronzo et al. (40) reported that bisphosphonates in bone-targeted agents could reduce the risk of bone metastases in some breast cancer patients, but not in lung or prostate cancer. Currently, no effective therapy specifically addresses prostate cancer with bone metastases (41, 42), and ADT remains the most common clinical approach for such patients before they progress to CRPC (43).

Our study underscores the importance of treatment strategies in the bone metastasis phase. Future research should compare different treatments—such as targeted therapy, chemotherapy, and radiotherapy—in hormone-sensitive patients with bone metastases to advance this field. In ANN models, obstructive symptoms and TNM stage emerged as the most significant features in FI. Obstructive symptoms also showed a significant difference in traditional statistical analysis (p < 0.001).

Research on the relationship between obstructive symptoms and prostate cancer progression remains limited. A 2013 study (44) noted that while bladder outlet obstruction is a major complication of locally advanced prostate cancer, it has minimal impact on prognosis—contrary to our findings. Maximilian Rom et al. (45) found that over half of CRPC patients exhibited detrusor overactivity, with some lower urinary tract obstruction symptoms related to overactivity and reduced bladder capacity. Further research is needed to clarify this association.

Additionally, our traditional statistical analysis indicated that non-progressive patients had higher average androgen levels than progressive patients, although this difference was not statistically significant. A meta-analysis of 25 studies (46) found that higher testosterone levels before ADT were associated with reduced mortality risk (HR = 0.58; 95% CI, 0.45–0.74; P < 0.0001). During ADT, lower testosterone levels were linked to reduced mortality risk (HR = 0.48; 95% CI, 0.28–0.81; P = 0.006) and lower progression risk (HR = 0.59; 95% CI, 0.46–0.77; P < 0.0001), consistent with our observations.

Finally, given the importance of treatment regimens highlighted in our ML analysis, more clinical studies with larger and more comprehensive datasets are needed to determine optimal treatment strategies for prostate cancer patients.

This study has several limitations. First, its retrospective design may introduce issues such as missing data, selection bias, and unmeasured confounding. To preserve data integrity, we did not impute missing values, resulting in a final sample size of 410. In settings with limited samples, numerous features, and imbalanced outcomes, models are prone to overfitting. Although we used GA for feature selection and some models incorporated SMOTE during that phase, our results indicated no substantial overfitting, supporting the validity of our approach. Nevertheless, larger sample sizes in future studies would help corroborate our findings.

Second, due to variable heterogeneity, we could not fully disentangle the influence of treatment during the hormone-sensitive phase on the overall model, as patients may respond differently to the same regimens. Subsequent studies should seek to minimize the impact of features with strong intercorrelations to improve model robustness.

Lastly, our study lacked external validation. The absence of multicenter, large-sample external validation limits the assessment of our model’s scalability. In future work, we plan to use public databases to validate and strengthen our findings.

## Conclusion

This study developed a machine learning-based predictive model for disease progression in HSPC patients, with internal validation confirming its clinical applicability. The ensemble model, particularly RF, demonstrated optimal predictive performance and serves as an effective tool for individualized risk stratification. Furthermore, the research established the independent prognostic impact of key factors including hormone-sensitive phase treatment strategies, HVD, and PSA levels, providing objective evidence to inform clinical decision-making.

## Data Availability

The original contributions presented in the study are included in the article/**Supplementary Material**. Further inquiries can be directed to the corresponding authors.
