# Supplementary material for: Machine learning approaches for predicting progression in hormone-sensitive prostate cancer patients
Source: Front Oncol. 2026 Feb 12;16:1704671. doi: 10.3389/fonc.2026.1704671 (PMC12935601; doi:10.3389/fonc.2026.1704671)
Supplement: Supplementary file 1 [file Table1.docx]

|  | kernel | C | gamma | degree | coef0 |
| --- | --- | --- | --- | --- | --- |
| SVM | Linear | 1 | 0.0 | 1 | 1 |

Table(S1)The parameters of SVM were adjusted
