# Supplementary material for: Machine learning approaches for predicting progression in hormone-sensitive prostate cancer patients
Source: Front Oncol. 2026 Feb 12;16:1704671. doi: 10.3389/fonc.2026.1704671 (PMC12935601; doi:10.3389/fonc.2026.1704671)
Supplement: Supplementary file 2 [file Table2.docx]

|  | n_estimators | max_depth | min_samples_leaf | min_samples_split | max_features | criterion |
| --- | --- | --- | --- | --- | --- | --- |
| DecisionTreer | - | 4 | 1 | 1 | 13 | gini |
| RandomForest | 35 | 4 | 5 | 5 | 10 | gini |

Table(S2)The parameters of DT and RF were adjusted
