# Supplementary material for: Machine learning approaches for predicting progression in hormone-sensitive prostate cancer patients
Source: Front Oncol. 2026 Feb 12;16:1704671. doi: 10.3389/fonc.2026.1704671 (PMC12935601; doi:10.3389/fonc.2026.1704671)
Supplement: Supplementary file 3 [file Table4.docx]

|  | n_estimators | max_depth | min_child_weight | nthread | scale_pos_weight | gamma | colsample_bytree | subsample | reg_alpha |
| --- | --- | --- | --- | --- | --- | --- | --- | --- | --- |
| XGboost | 54 | 6 | 5 | 1 | 1 | 0.29 | 0.4 | 0.4 | 0.1 |
|  | learning_rate |  |  |  |  |  |  |  |  |
|  | 0.2 |  |  |  |  |  |  |  |  |
| Early stopping | eval_metric | eval_set | verbose | early_stopping_rounds |  |  |  |  |  |
|  | logloss | eval_set | True | 300 |  |  |  |  |  |

Table(S4)The parameters of XGboost were adjusted
