# Supplementary material for: Machine learning approaches for predicting progression in hormone-sensitive prostate cancer patients
Source: Front Oncol. 2026 Feb 12;16:1704671. doi: 10.3389/fonc.2026.1704671 (PMC12935601; doi:10.3389/fonc.2026.1704671)
Supplement: Supplementary file 4 [file Table5.docx]

| Attribute name | Attribute description |  |
| --- | --- | --- |
| Age | Age in years |  |
| Obstruction symptom | The value with obstruction is 1, and the value without obstruction is 0 |  |
| Hematuria symptom | The value with hematuria is 1, and the value without hematuria is 0 |  |
| Whether to touch the nodules | If the anal digital examination has touched nodules, it is 1, and the untouched nodules are 0 |  |
| Pathological type | 1 for adenocarcinoma, 2 for intraductal carcinoma, 3 for urothelial carcinoma, 4 for mucinous carcinoma, 5 for neuroendocrine carcinoma and 6 for mixed carcinoma |  |
| Prostatic volume(cm) | The volume of the prostate in cm^3^ |  |
| fPSA at first visit | The fPSA value of the patient at first diagnosis |  |
| TPSA at first visit | The TPSA value of the patient at first visit |  |
| f/tPSA | The ratio between fPSA and TPSA |  |
| Bone metastases at first visit | The presence of bone metastasis is 1, and the absence of bone metastasis is 0 |  |
| Visceral metastasis at first visit | If there is an organ transfer, it is 1, and otherwise it is 0 |  |
| Tumor burden | According to the criteria for the diagnosis and Treatment of Urology and Andrology Diseases in China 2022, the transfer load is 1 for high and 0 for vice versa |  |
| T1-2 | Whether the TNM stage of the patient is within this interval , 1 or 0 |  |
| T3-4 | Ibid |  |
| N0 | Ibid |  |
| N1 | Ibid |  |
| M0 | Ibid |  |

Table(S5)Feature subsets of different patient datasets
