# Supplementary material for: Machine learning approaches for predicting progression in hormone-sensitive prostate cancer patients
Source: Front Oncol. 2026 Feb 12;16:1704671. doi: 10.3389/fonc.2026.1704671 (PMC12935601; doi:10.3389/fonc.2026.1704671)
Supplement: Supplementary file 5 [file Table7.docx]

|  | neurons | activation | kernel_initializer | Dropout | epochs |
| --- | --- | --- | --- | --- | --- |
| Neural Network | 64 | Tanh/sigmoid | glorot_uniform | 0.1 | 150 |
|  | batch_size | verbose | validation_split | Loss | metrics |
|  | 32 | 1 | 0.2 | binary_crossentropy | accuracy |
|  |  |  |  |  |  |
|  |  |  |  |  |  |
| Early stopping | restore_best_weights | monitor | patience |  |  |
|  | True | val_loss | 10 |  |  |

Table(S7)The parameters of ANN were adjusted
